# Separation and Recovery of Fine Particles from Waste Circuit Boards Using an Inflatable Tapered Diameter Separation Bed

**DOI:** 10.1155/2014/843579

**Published:** 2014-10-15

**Authors:** Chenlong Duan, Cheng Sheng, Lingling Wu, Yuemin Zhao, Jinfeng He, Enhui Zhou

**Affiliations:** China University of Mining & Technology, No. 1, Daxue Road, Quanshan District, Xuzhou 221116, China

## Abstract

Recovering particle materials from discarded printed circuit boards can enhance resource recycling and reduce environmental pollution. Efficiently physically separating and recovering fine metal particles (−0.5 mm) from the circuit boards are a key recycling challenge. To do this, a new type of separator, an inflatable tapered diameter separation bed, was developed to study particle motion and separation mechanisms in the bed's fluid flow field. For 0.5–0.25 mm circuit board particles, metal recovery rates ranged from 87.56 to 94.17%, and separation efficiencies ranged from 87.71 to 94.20%. For 0.25–0.125 mm particles, metal recovery rates ranged from 84.76 to 91.97%, and separation efficiencies ranged from 84.74 to 91.86%. For superfine products (−0.125 mm), metal recovery rates ranged from 73.11 to 83.04%, and separation efficiencies ranged from 73.00 to 83.14%. This research showed that the inflatable tapered diameter separation bed achieved efficient particle separation and can be used to recover fine particles under a wide range of operational conditions. The bed offers a new mechanical technology to recycle valuable materials from discarded printed circuit boards, reducing environmental pollution.

## 1. Introduction

Over the past 20 years, electric and electronics equipment (EEE) production has become a fast growing industry, maximizing the benefits of technological innovation and scientific process [1]. The usage life of many kinds of EEE has been substantially shortened due to electronics industry advancements, attractive consumer designs, and intense marketing [2]. The combination of rapid industry growth and shorter use cycles has led to a significant increase in waste electric and electronic equipment (WEEE), and WEEE is considered the fastest-growing component of solid waste in the wide world [3, 4].

Printed circuit boards, the foundation of electronic industry, are an essential part of almost all EEE, leading to a large volume of waste once discarded. Waste printed circuit boards (WPCB) hold great potential value, because they contain twenty kinds of nonferrous and rare metals. Unfortunately, WPCB also contain a number of heavy metals, including lead, cadmium, mercury, and nickel, as well as brominated flame retardants. All of these present potential dangers to the soil, to the environment, and to biological organisms [5–7]. Reutilizing WPCB is an important research topic, because developing an efficient, nonpolluting, low-cost processing technology for recycling WPCB may reduce pollution and save resources. Current primary methods of recycling WPCB include hydrometallurgy, pyrometallurgy, biometallury, pyrolysis, and mechanical process [8–10]. With the gradual decrease in precious metals used in WPCB, more attention is being paid to mechanical processes, with their better environmental properties, high efficiencies, and easier operation. Once concentrated, recovered metals can be handled using chemical methods for further separation and purification. The main components of nonmetallic concentrate from WPCB are resin and glass fibers, which may be recycled and reutilized as fillers, waterproofing materials, or building materials [11].

The mechanical recycling process for WPCB is driven by differences in the physical characteristics of the materials, including density, magnetic susceptibility, and electric conductivity [8, 12, 13]. Corona electrostatic separation has proven to be suitable for separating metals from pulverized WPCB with particle sizes between 0.6 and 1.2 mm. Xu et al. proposed an integrated cyclone air separation-corona electrostatic separation process to treat WPCB, and an industrial-scale production line with the capacity of 500 kg/h–1000 kg/h has been established [14, 15]. However, because particles can easily be repelled, appealed, or bunched together when the size drops, effective corona electrostatic separation is difficult to achieve at a particle size of less than 0.6 mm [16].

In China, water table separation is another key technique separating particles with the size range of 0.5–2 mm from WPCB. In fact, over one hundred plants use water table separation to recycle WPCB in China [3, 9]. However, for −0.25 superfine particles, over 70% of the metals will experience loss to tailings, due to the mismatch of the setting velocity with water table separation technology. The low separation efficiency for superfine particles (−0.25 mm) has emerged as an obstacle to the mechanical processes involved in recycling WPCB. As such, it is critical to develop a technique for treating the superfine particles of WPCB.

A laboratory-scale tapered diameter separation bed (TDSB) was designed using computational fluid mechanics to separate metal concentrate from fine particles of WPCB. The research was conducted at the China University of Mining & Technology during the period of March 2006 until 2012. For less than 0.074 mm superfine WPCB particles, the metal recovery rate using the TDBS ranged from 48.23% to 93.42% and the separation efficiency ranged from 60.59% to 77.63%. This result suggested that the lower separation limit of particle size using the TDSB may be close to zero [17–20]. However, as separation time increases, material accumulation in the TDSB deteriorates separation efficiency and equipment reliability decreases. To improve the longer-range reliability of the TDSB, we redesigned it as an inflatable TDSB (ITDSB) to separate the fine particles of WPCB at a laboratory scale. A density-dominant separation was enhanced and the separation efficiency was improved greatly by the secondary separation. Fine particles (−0.5 mm) of WPCB can be recovered effectively using ITDSB. This technique will prevent environmental pollution from WPCB and facilitate the efficient recovery of resources.

## 2. Materials and Methods

### 2.1. Sample Preparation and Characterization

This experiment used crushed WPCB without electronic elements, obtained from a local PCB factory. The purpose of the crushing was to liberate copper-based metals from the cladding materials and composite laminates (such as resin, fiberglass, and plastics), because copper liberation is a key indicator for successful downstream separation. For the 0.5 mm particle size, the metal liberation rate was over 95% for both the baseplate and the molectron. For this ITDSB research, we decided to use particles of 0.5 mm size from WPCB after considering the degree of metal liberation, the particle distribution ratio of the metal, and options for simplifying the technology. This resulted in three different size particles for the experiment. Sample I is 0.5–0.25 mm, Sample II is 0.25–0.125 mm, and Sample III is −0.125 mm. Metals identification was measured using a D8 ADVANCE XRD (Bruker, German). The metal content of Samples I, II, and III was 5.02%, 4.21%, and 3.01%. The metals content of WPCB decreases as the particle size decreases.

### 2.2. Inflatable Tapered Diameter Separation Bed

**Figure 1:**
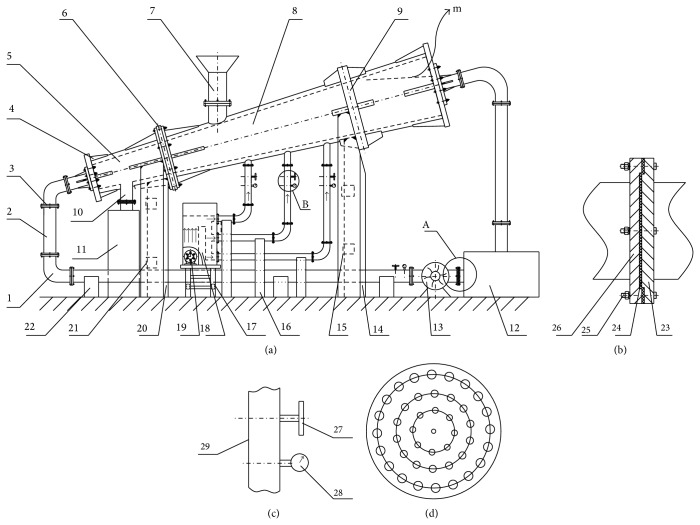
(a) ITDSB schematic. (b) Enlarged view of area A. (c) Enlarged view of area B. (d) Schematic of the hydraulic distributor.* Note.* 1—Elbow pipe, 2—straight pipe, 3—flat flange, 4—stiffener board, 5—front-end of TDSB, 6—hydraulic distributor, 7—feeding inlet, 8—back-end of TDSB, 9—bearing ring, 10—discharge port, 11—concentration tank, 12—circulating water tank, 13—circulating water pump, 14—the middle brace of the ITDSB back end, 15—bilateral brace, 16—straight pipe brace, 17—bellows block, 18—bellows, 19—air blower, 20—middle brace, 21—bilateral brace, 22—straight pipe brace, 23—convex flange, 24—filter cloth, 25—bolt, 26—concave flange, 27—valve, 28—flow meter, 29—fluid pipeline, and m—air-water interface.

Based on fluid dynamic calculations, the problematic settling trends, and the theory of inclined flow separation, we redesigned a new laboratory-scale separator ITDSB. The ITDSB has a top diameter of 200 mm, a bottom diameter of 120 mm, and a height of 800 mm. To observe particle segregation, a special plexiglass sheet was used to form the separation bed body.  Figure 1(a) displays a schematic diagram of ITDSB. First, a pump circulates water from a tank into the bottom of ITDSB. Second, slurry, which has been homogenized in a beater, enters the upper part of the ITDSB. Low-density particles reflux into the circulation water tank with the flowing water, where it is separated by a filter cloth.  Figure 1(b) displays a partial enlargement of the filter, through which the water can be recycled. The high-density particles are extracted from a different discharge outlet, driven by hydraulic conditions. The gas flow enters the bed through the air blower.  Figure 1(c) is a partial enlargement of air supply control system. The gas flow loosens deposited materials, which flow through the boundary layer and enter the separation area. Then, the deposited materials are separated again under the effect of the uprising flow. Separation efficiencies improve significantly through a secondary separation. To improve fluid homogeneity and stability, a new distributor was designed, with an aperture ratio and pore diameter, which increase from the center to the edge of the distributor (see Figure 1(d)). The new distributor is easier to manufacture and helps produce a stable, rising fluid. Moreover, it prevents distributor hole choking and also relieves liquid-solid back mixing. The separation bed body was tilted to increase the velocity gradient of the rising water and broaden the minimum separation limit.

### 2.3. Evaluation Index

To evaluate the separation effect, the variables of metal recovery (*R*) and separation efficiency (SE) were adopted and expressed as follows:

(1) $$\begin{array}{c} R=\frac{c\beta }{c\beta +t\theta }\times 100\%,\\ \text{SE}=\frac{\beta \left(\alpha -\theta \right)}{\alpha \left(\beta -\theta \right)}\times 100\%. \end{array}$$

In these equations, *α*, *β*, and *θ* represent the metal content of feeding, concentrate, and tailing, respectively. The variables *c* and *t* represent yield of concentrate and tailing, respectively.

## 3. Results and Discussion

### 3.1. Force Analysis of Particles at Different Position of ITDSB

**Figure 2:**
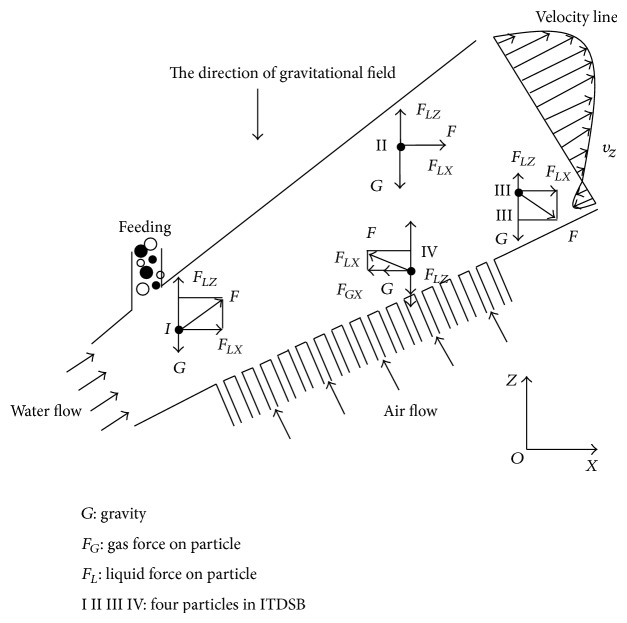
Sketches depicting how changes in diameter and forces impact particles.

Figure 2 illustrates the force and motion of particles in different positions and velocity field of the cross section below the air-flow interface in ITDSB. First, looking at Particle I, the bed's diameter is quite small at that position, leading to a higher water velocity and a turbulent flow field. Particles in this field are far away from gas tubes, so the gas flow has less effect on particle motion, and the interaction between liquid and solid phases is the main force. The direction of force *F*_*LZ*_, which is the component force of *F* on the *z*-axis, moves upward and it is far larger than gravity *G*. As such, particles move upward and forward under the effect of those forces. With the liquid motion, the bed size becomes increasingly larger, and the velocity of the liquid decreases. *F*_*LX*_ and *F*_*LZ*_ also gradually decline, while *G* is steady. When the value of *F*_*LX*_ is equal to *G*, the force in the *Z* direction of particles reaches a balance, and the total force *F* equals *F*_*LX*_.

Next, turning to Particle II, after the particle reaches a force balance in *z* axis, particle movement varies at different positions. We analyzed ITDSB flow field distribution, as well as the water flow velocity in the *Z* direction. Figure 2 illustrates that the particle position is above the velocity line with the highest speed value. Due to the continuous motion of the particles and the enlargement of cross section, the particle velocity in the *Z* direction decreases. The velocity of particle approaches the velocity line with the highest speed value which leads to the increase in the particle velocity. Particles are dragged to the outlet by inertia and *F*_*LZ*_ before passing the maximum velocity line. When particles pass that velocity line, velocity in the *Z* direction declines, and *F*_*LZ*_ decreases considerably, as well as *F*_*LX*_. Particles are dragged to the bottom wall under the effect of joint forces. Particles sink sharply when *G* is constant, as indicated in the sketch above at the point marked by particle III. According to the ITDSB velocity distribution analysis, back flow occurs at the outlet near the lower wall. Particles entering the area of back flow move along the wall towards the inlet. When a particle approaches the air tube area, it is pushed away from the original direction by the force of the incoming air. Gas force in the *Z* direction is far larger than *G*, which leads to the particle's upward motion, as indicated in the sketch above at the point marked by particle IV.

### 3.2. Movement Trajectories of Different Particles

**Figure 3:**
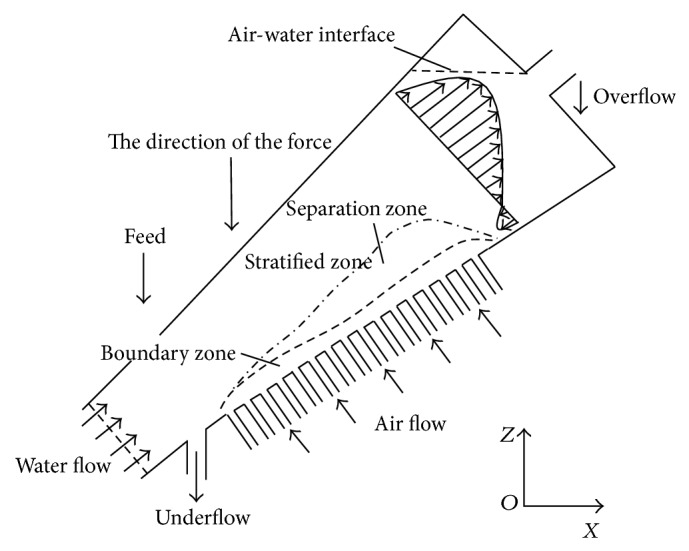
Flow field distribution in ITDSB.

As Figure 3 shows, the ITDSB can be divided into three regions, including the stratified zone, boundary zone, and separation zone. The particles are loosened and stratified by the turbulent vortex formed by the flow at the bottom of the bed, removing the particles' primary stratification. Due to restrictions of the bed diameter, the particles initially stratified, resulting in intermediate density particles sinking down to the lower wall. This forms a boundary zone, which deteriorates the separating effect. To compensate for this, gas flow is added to the bed to loosen the particles, forcing them out of the boundary zone into the separation zone. The particles are then separated in the rising water flow.

**Figure 4:**
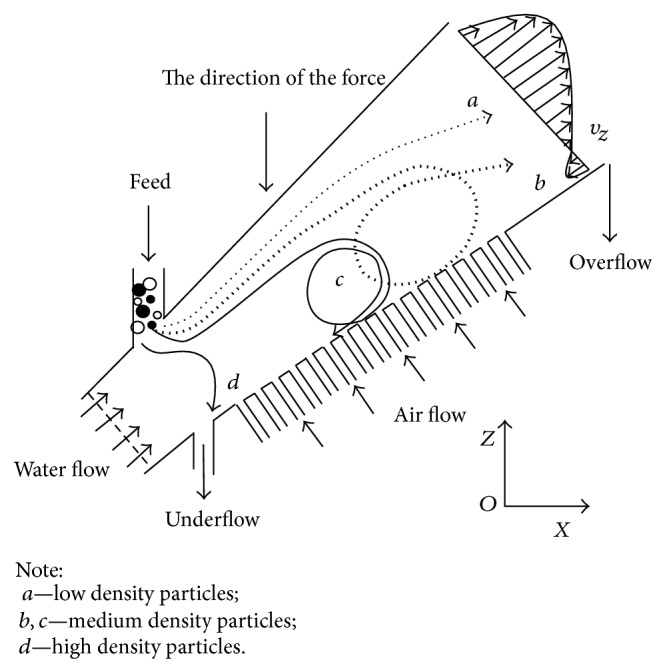
Movement trajectories of different particles.

From the top to the bottom of the ITDSB, the particle density gradually increases. Particles with different terminal velocities, based on the density, size, and shape of the particle, are separated in the ITDSB flow field.  Figure 4 shows how particle velocity and direction depend on the settling velocity of the particles and the water velocity. When the particles are fed into the ITDSB, the water flow rises; thus light particles with lower density and smaller size move to the top area as overflow. In the figure, this is shown by trajectory “*a*.” Trajectory “*d*” shows that heavy particles with a higher density and larger size will travel through the whole bed and become concentrates. Particles with intermediate density and size are mainly solid phase materials in the liquid-solid bed. In the sorting process, high-density particles sink, which influences the separation effect. Air flow is added into the ITDSB to loosen accumulated particles and generate a secondary separation. Trajectories of “*b*” and “*c*” show the separation process of the accumulated high-density and low-density particles. Particle “*b*” ultimately becomes the tailing and particle “*c*” flows out from the bottom outlet under the effect of water and gas flow. The expanding tubular structure of the separation bed body causes a decreasing water velocity as the flow proceeds along the axis. A density-dominant separation was reinforced, and the separation efficiency improves through secondary separation.

### 3.3. Separation Performance of ITDSB

The main factors influencing ITDSB separation performance are water feed rate, gas flow rate, tube obliquity, and slurry concentration. Effective separation could be achieved with a tube obliquity range of 30–60° and a slurry concentration range of 200–400 g/L. In this experiment, the tube obliquity was set at 40°, the slurry concentration was 300 g/L, the water feed rate was set at 0.5, 1.0, and 1.5 m^3^/h, and the gas flow rate was set at 0.4, 0.8, and 1.2 m^3^/h. Experimental data are shown in Table 1. During these trials, for 0.5–0.25 mm sized materials, metals recovery ranged from 87.56 to 94.17% and separation efficiency ranged from 87.71 to 94.20%. For 0.25–0.125 mm sized particles, metals recovery ranged from 84.76 to 91.97% and separation efficiency ranged from 84.74 to 91.86%. For −0.125 mm superfine materials, metal recovery ranged from 73.11 to 83.04% and separation efficiency ranged from 73.00 to 83.14%. These experiments demonstrate that ITDSB can be used to achieve effective separation and recover fine WPCB particles under a wide range of operational conditions. With a decrease in particle size, the separation effect worsens. ITDSB allows for density driven separation, which leads to terminal velocities separation, driven by the size, shape, and density of the particles. The metals content of 0.125 mm samples is only 3.01%. However, the metal content can be concentrated up to 9.72% using ITDSB; this concentrate can then be handled using chemical methods to achieve further separation and purification. Superfine products with a size range of 0.0125 mm were recovered effectively from WPCB using ITDSB, offering a new technique to recycle resources and reduce environmental pollution.

## 4. Conclusions

A new inflatable tapered diameter separation bed was designed to study particle separation mechanisms and flow, with the goal of increasing the efficiency of heavy metal separation from discarded circuit boards. Effective separation results were achieved using the new technology. For 0.5–0.25 mm circuit board particles, metal recovery rates reached 94.17% and separation efficiency reached 94.20%. For 0.25–0.125 mm particles, metal recovery rates ranged from 84.76 to 91.97% and separation efficiency ranged from 84.74 to 91.86%. For superfine products (−0.125 mm), metals recovery and separation efficiency reached 83.04% and 83.14%, respectively.

When considering force and motion dynamics, particles with different sizes and densities could be separated efficiently by ITDSB due to its inflatable structure and flow field. Ultimately, ITDSB allows density driven separation, which facilitates terminal velocity separation driven by the size, shape, and density of the particles.

## Figures and Tables

**Table 1:** Experimental results from respectively separating 0.5–0.25 mm, 0.25–0.125 mm, and −0.125 mm WPCB.

Size (mm)	Metal content of WPCB (%)	Water feed rate (m^3^/h)	Gas flow rate (m^3^/h)	Product	Yield (%)	Metal content (%)	Recovery (%)	Separation efficiency (%)
0.5~0.25	5.02	0.5	0.4	Concentrate	52.12	9.05	94.17	94.20
				Tailing	47.88	0.61		
		1	0.4	Concentrate	40.32	11.21	90.04	90.01
				Tailing	59.68	0.84		
		1.5	0.4	Concentrate	35.65	12.33	87.56	87.71
				Tailing	64.35	0.96		
		0.5	0.8	Concentrate	44.28	10.57	90.59	90.46
				Tailing	55.72	0.84		
		0.5	1.2	Concentrate	36.41	12.22	88.63	88.73
				Tailing	63.59	0.89		

0.25~0.125	4.21	0.5	0.4	Concentrate	46.54	8.32	91.97	91.86
				Tailing	53.46	0.64		
		1	0.4	Concentrate	38.1	10.06	91.04	91.03
				Tailing	61.9	0.61		
		1.5	0.4	Concentrate	30.72	12.05	87.89	87.99
				Tailing	69.28	0.73		
		0.5	0.8	Concentrate	42.16	9.01	90.23	90.25
				Tailing	57.84	0.71		
		0.5	1.2	Concentrate	33.76	10.57	84.76	84.74
				Tailing	66.24	0.97		

−0.125	3.01	0.5	0.4	Concentrate	40.25	6.21	83.04	83.14
				Tailing	59.75	0.85		
		1	0.4	Concentrate	30.78	7.98	81.6	81.86
				Tailing	69.22	0.79		
		1.5	0.4	Concentrate	22.64	9.72	73.11	73.00
				Tailing	77.36	1.05		
		0.5	0.8	Concentrate	32.01	7.45	79.23	78.96
				Tailing	67.99	0.93		
		0.5	1.2	Concentrate	25.67	9.16	78.12	78.01
				Tailing	74.33	0.89		
